# Assimilable organic carbon (AOC) determination using GFP-tagged *Pseudomonas fluorescens* P-17 in water by flow cytometry

**DOI:** 10.1371/journal.pone.0199193

**Published:** 2018-06-14

**Authors:** Peng Tang, Jie Wu, Hou Liu, Youcai Liu, Xingding Zhou

**Affiliations:** 1 School of Life Sciences and Chemical Technology, Ngee Ann Polytechnic, 599489, Singapore, Singapore; 2 College of Chemistry and Chemical Engineering, Central South University, Changsha 410083, PR China; Hunan University, CHINA

## Abstract

One of the newly developed methods for Assimilable organic carbon (AOC) determination is leveraged on the cell enumeration by flow cytometry (FC) which could provide a rapid and automated solution for AOC measurement. However, cell samples staining with fluorescence dye is indispensable to reduce background and machine noise. This step would bring additional cost and time consuming for this method. In this study, a green fluorescence protein (GFP) tagged strain derived of AOC testing strain *Pseudomonas fluorescens* P-17 (GFP-P17) was generated using Tn5 transposon mutagenesis. Continuous culture of this mutant GFP-P17 showed stable expression of eGFP signal detected by flow cytometry without staining step. In addition, this GFP-P17 strain displayed faster growth rate and had a wider range of carbon substrate utilization patterns as compared with P17 wild-type. With this strain, the capability of a new FC method with no dye staining was explored in standard acetate solution, which suggests linear correlation of counts with acetate carbon concentration. Furthermore, this FC method with GFP-P17 strain is applicable in monitoring GAC/BAC efficiency and condition as similar trends of AOC level in water treatment process were measured by both FC method and conventional spread plating count method. Therefore, this fast and easily applicable GFP-P17 based FC method could serve as a tool for routine microbiological drinking water monitoring.

## Introduction

Growth of bacteria in drinking water treatment, distribution and storage systems can result in deterioration of water quality, violation of water quality standards and increased operating expenses. Bacterial growth or regrowth can result from viable bacteria surviving the disinfection process and utilizing nutrients in the water or biofilm to sustain growth[1]. Microbial activity in the distribution system largely depends on the nutrients particularly carbon source. Controlling microbial activity in water treatment and distribution system is of great importance to prevent water quality deterioration resulting in non-compliance with regulations, consumer complaints and disease. Not all organic compounds are equally susceptible to microbial decomposition and the fraction that provides energy and carbon for bacterial growth has been called assimilable organic carbon (AOC) [2]. The AOC is comprised of a wide variety of low-molecular-weight organic carbon molecules such as sugar, organic acids and amino acids and represents only a small fraction (< 10%) of the total organic carbon (TOC)[3]. The AOC is usually considered as one of the main indicators of biological water stability and is a critical parameter for drinking water treatment and distribution processes[4, 5]. Recently, the AOC concept has also been extended to measure microbial growth potential for environmental samples such as soil water extracts [6–8], seawater [9–11] and reclaimed water[12, 13].

Conventional AOC analysis was originally developed by Dutch researcher Van der Kooij and co-workers[14–17] and later adapted by others as stated in the Standard Methods[17]. This method basically contains two stages: cell culturing and cell enumeration. In the cell culturing stage, the water sample is inoculated with two water representative strains *Pseudomonas fluorescens* P-17 (P17 in short) and *Spirillum* sp. NOX (NOX in short), and incubated at 15°C for 9 days to reach the stationary phase. On days 7, 8 and 9, culture cell enumeration is determined by traditional plating counting on nutrient agar, which required another 3–5 days. Finally the cell numbers of the two strains are converted to AOC amount by previously derived empirical yield values[17]. Obviously, this spread plate counting (SPC) based method is tedious, labour-intensive and notably time-consuming (>14 days). Thus, a rapid and easily applicable AOC testing method is essential for an efficient water treatment system and water bio-stability monitoring.

Over the last decades, various AOC method development has been explored, which mainly focused on three aspects: the selection of inoculum, the optimization of inoculation and incubation, and the evolution of bacterial growth measurements[18]. The last aspect, due to its significance for reducing cell enumeration time, received most attention recently. These new enumeration methods include examples like plate count[19, 20], turbidity method[15], ATP luminescence[13, 16], bioluminescence[9, 13, 21–23]. In the last decade, researchers have made much efforts to explore flow cytometry as a new AOC determination method. Coupled with fluorescent staining, flow cytometry has emerged as a new tool to challenge conventional heterotrophic plate counts for routine microbiological drinking water monitoring [24, 25]. In AOC determination, Tomas Egli group firstly demonstrated that cell enumeration by flow cytometry is a rapid and straightforward method and also able to obtain kinetic data to provide much clearer insight into the water growth potential[26]. Other groups further developed flow cytometry method by inoculum variation, fluorescence staining procedure optimization and flow cytometer gating set-up[5, 27–29]. However, all these newly developed flow cytometry methods require expensive fluorescence compounds to stain the bacteria prior to analysis and the staining procedure also adds additional time (15–20 mins) and works. Here we report a green fluorescence protein (GFP) tagged *Pseudomonas fluorescens* P-17 (GFP-P17 in short) strain and demonstrated that the above issues could be overcome by this new strain.

## Materials and methods

### Preparation of organic carbon-free glassware

Glassware were prepared as described in Standard Methods[17]. The vials (40mL) were first washed with detergent and thereafter rinsed thrice with Mini-Q water. Then these vials were soaked in 0.1N HCl overnight and again rinsed with Mini-Q water three times. The vials were subsequently heated in a muffle oven at 550°C for 5.5h to remove all trace organics. The screw caps were soaked in a 10% sodium persulfate solution at 60°C for at least 1h and then rinsed with Mini-Q water and finally air-dried.

### Inoculum preparation

The culture P17 (ATCC49642) or GFP-P17 was stored in 20% glycerol at -70°C. When needed, the culture was recovered to a freshly prepared R2A agar plate. After 3 days incubation, several single colonies were inoculated into a sterile ground-glass-stoppered Erlenmeye flask containing 100 mL of autoclaved Milli-Q water with 1 mg/L of acetate-C and mineral salts (0.1 mM K2HPO4, 1.4 mM NH4Cl and 1.4 mM KNO3). The flask was incubated at 25°C for 6 days to ensure that all residual organic carbon had been consumed. These inoculums were stored at 4°C for not more than 6 months and determined the cell concentration before each AOC assay.

### Construction of GFP-P17 strain

A mini-Tn5 transposon system (Biomedal Life Science, Spain) was chosen to construct P17 GFP tagged strain[30]. The procedure is described as in the system manual. Briefly, GFP encoding region with a constitutive promoter was cloned and inserted into the Not I site of pUTmini-Tn5 Cm plasmid to construct the recombinant plasmid pUTmini-Tn5 Cm GFP in *E*. *coli* strain S17-1λpir. The recombinant plasmid isolated from *E*. *coli* strain S17-1λpir was transferred into P-17 by electroporation using a Bio-Rad MicroPulser. Selection of GFP tagged strains was performed by colony PCR screening and restriction enzyme digestion. The expression of GFP was examined using flow cytometry.

### AOC Testing solutions

Water samples obtained from a local water treatment plant (primary filter outlet, ozone outlet and GAC outlet) were firstly pretreated by adjustment of pH to 6.8 to 7.2 with 0.1 M of NaOH or 0.1 N HCl followed by pouring 40ml of the water samples into glassware vials and pasteurizing in 70°C water bath for 30 minutes. Mineral salts was added into the samples as recommended. After cooling down, the water samples were inoculated with P17 or GFP-P17 at final concentration of 500 pfu/mL followed by incubation at 25°C for 3 days before analysis. Triplicate vials were prepared for each test condition.

The AOC assay was also performed on testing solution comprised of sodium acetate (0–1000 µg/L as C) in autoclaved ultrapure water with mineral salts to obtain yield value.

### Flow cytometry measurements

Flow cytometry measurements were performed using Beckman Coulter cytoFlex flow cytometer equipped with a laser emitted at fixed wavelength of 488nm. Fluorescence intensity was collected at FITC = 530+20nm while sideward and forward scattered light intensities were collected as well. The FCM is equipped with volumetric counting hardware, calibrated to measure the number of particles in 60 µl of 500 µl sample. Measurements were performed at pre-set flow rate of 60 µl/min. All data were processed with the Beckman Flow software, and electronic gating was used to separate positive signals from instrument and water sample background. A threshold value of 500 was applied on the green fluorescence channel. Unless stated otherwise, the instrument settings and electronic gates were kept the same for all samples in order to achieve comparable data.

### AOC analysis by spread plate counting

Analysis was carried out as described by Standard Methods[17]. In short, in the enumeration stage, bacterial suspensions were subjected to 10-fold dilutions with phosphate buffer saline (PBS) as needed, spread onto R2A agar plates, incubated at 25°C for 2–3 days. The AOC concentration was calculated from the maximum concentration of each of the P17 and NOX strains with the conversion factors of 4.6 x 10^6^ cfu/µg-C for strain P17 and 1.2 x 107 cfu/µg-C for strain NOX.

### Substrate utilization analysis

A BIOLOG GN2 plate assay[31] was adopted to analyze and compare the substrate utilization pattern for wild type P17 and GFP-P17. Total 95 low molecular weight carbons including amino acids, sugars and organic acids were tested. The assay was done as described in the manual and the results was observed by 570 nm absorbance.

### Statistical analysis

The data analysis package in Microsoft excel was used to perform regression, hypothesis testing, evaluating 95% CI and other statistical analysis. Paired t-test was used to evaluate the significance of the AOC results for samples from the local water treatment processes, at the level p < 0.05.

## Results

### Generation and characterization of *P*. *fluorescens* P-17 mutant tagged with GFP

**Fig 1:**
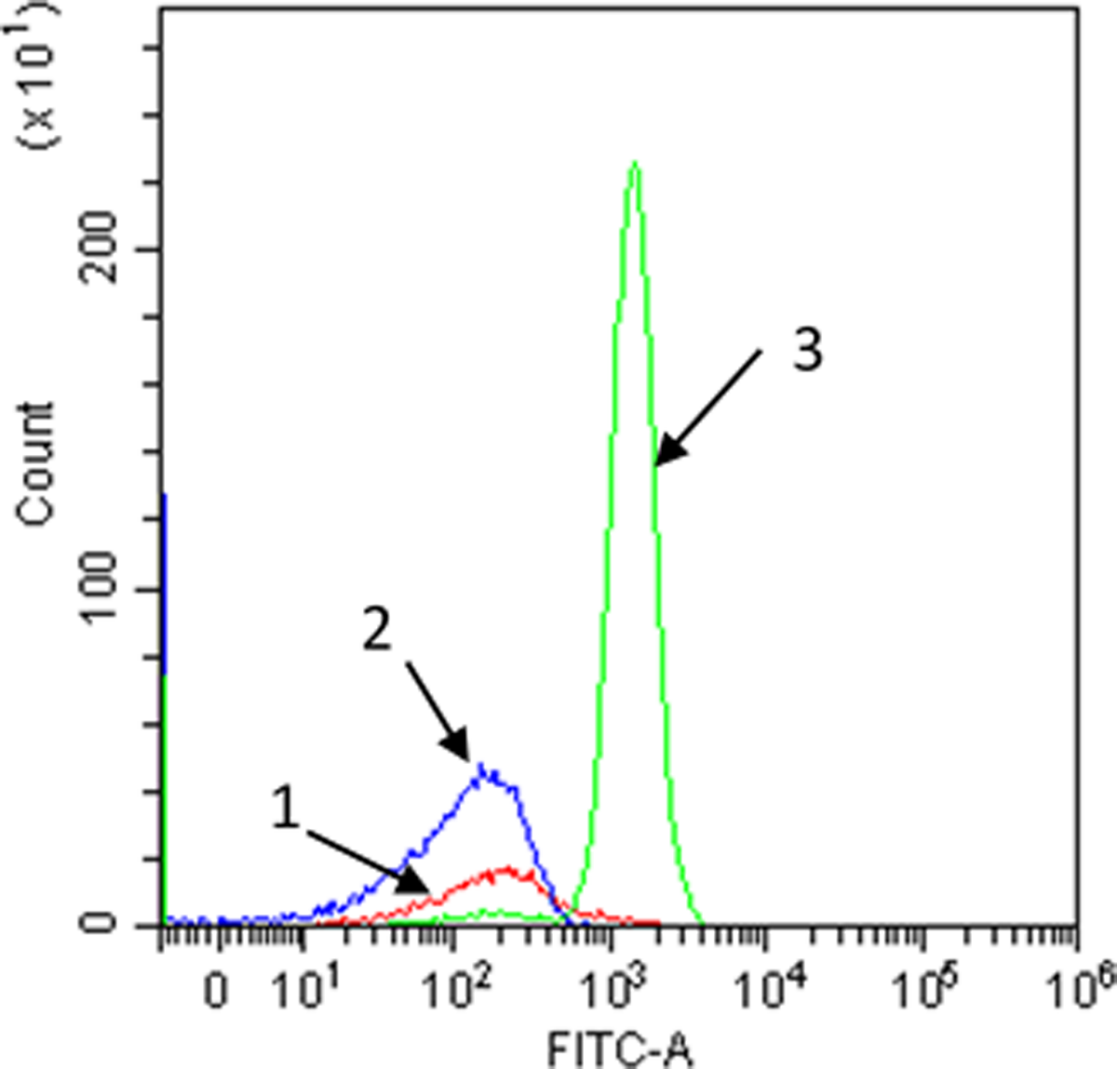
Representative fluorescence histogram data of GFP-P17 and P17 in water. The x-axis shows green (FITC) fluorescence intensity, and the y-axis shows the number of events recorded for a corresponding fluorescence intensity. 1, water sample without cells; 2, water sample with P17; 3, water sample with GFP-P17.

The AOC testing bacteria *P*. *fluorescens* P-17 was subjected to transposon mutagenesis for stable insertion of GFP operons into their genomes using electroporation method. Forty independent clones of mutated P-17 were screened for GFP insertion and expression. Colony PCR results shows that there are GFP DNA fragments in these colonies. To further select the brightest mutant with the expression of GFP from these positive colonies, flow cytometry analysis was performed. As shown in Fig 1, one mutant named GFP-P17, was obtained with strong green fluorescence intensity compared with wild type cells (Fig 1). In addition, with continuous subculture of the GFP-P17 cells, there is stable expression of GFP in the cells after 20 passages, indicating the genome insertion is stable.

### Growth of GFP-P17 and expression of GFP in GFP-P17

**Fig 2:**
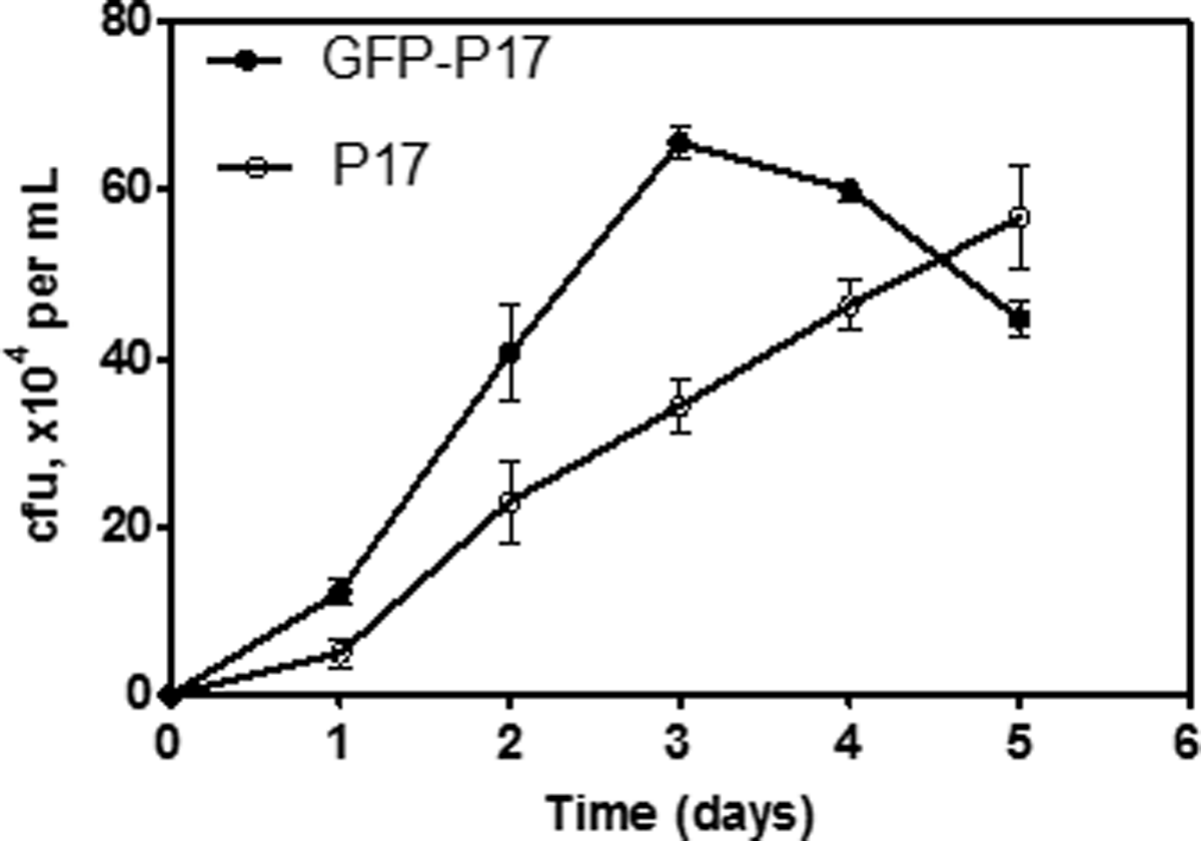
Batch growth curves of GFP-P17 and P17. Growth conditions: 100 µg/L of acetate carbon with supplement of mineral salts. Error bars indicate standard deviation on triplicate samples.

**Fig 3:**
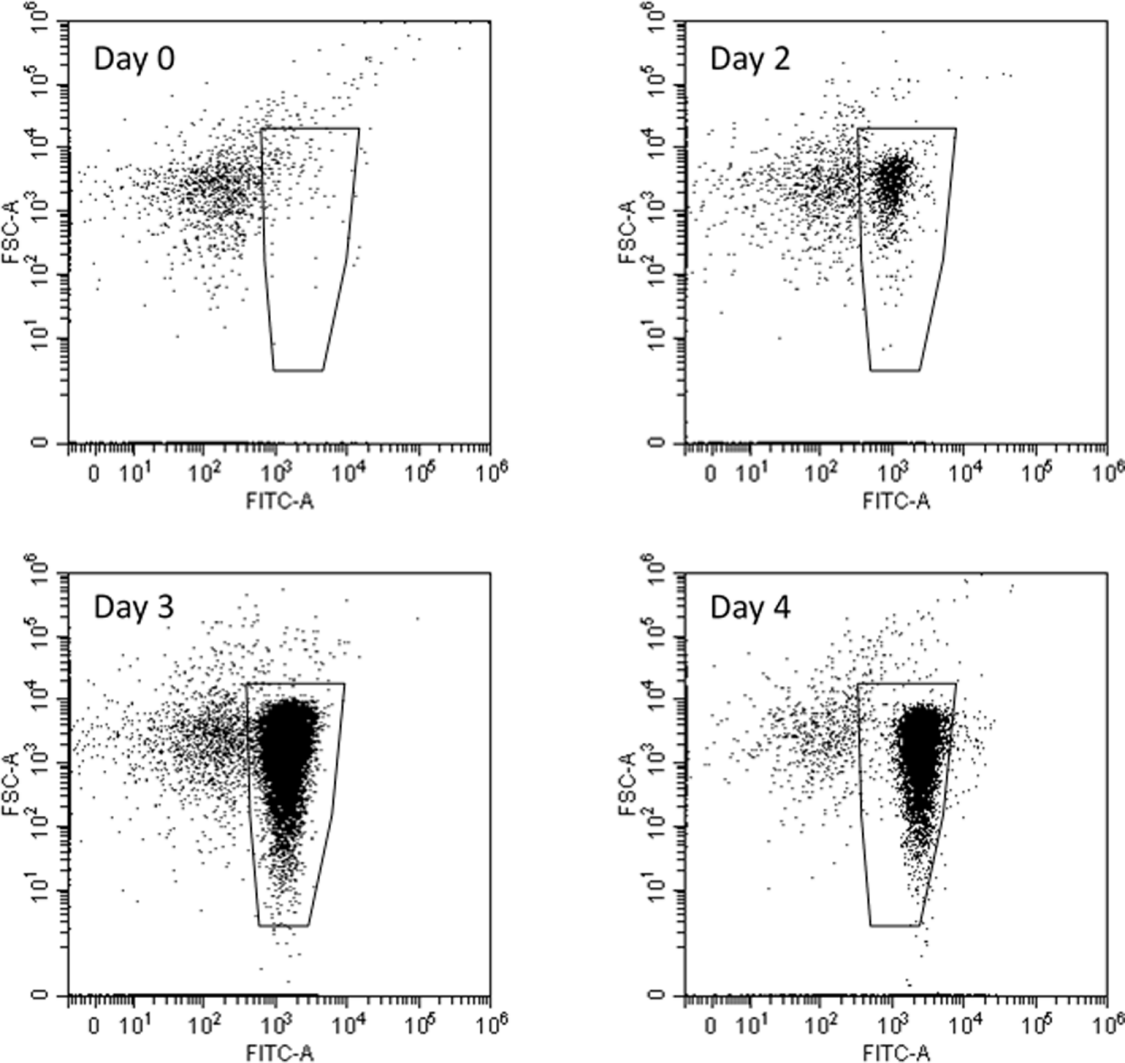
Dot plots for GFP-P17 cells grown on water of 100 µg/L of acetate carbon with supplement of mineral salts. Enumeration gates are drawn to distinguish cells from background. Data was recorded in one minute. Cell counts: Day 0, 457/mL; Day 2, 3647/mL; Day 3, 80737/mL; Day 4, 61508/mL.

Fig 2 shows that GFP-P17 grows to the onset of stationary phase on day 3 based on spread plate counting, this is even faster than the growth rate of the wild type P17 under the same testing condition. However the maximum cell concentration of GFP-P17 reduces slightly. The GFP expression signal is detected since day 2 by flow cytometry counting at green channel, and cell number reaches the peak on day 3 and drops from day 4 onwards (Fig 3).

### Comparison of carbon substrate utilization pattern between P17 and GFP-P17

One major consideration for GFP tagged strain is the carbon source utilization pattern. Previous study reported that P17 wild type strain was able to utilize a wide range of low molecular carbon sources such as organic acids, amino acids, and carbohydrates [2]. To investigate impact of GFP insertion into genome of P17 on its utilization of carbon sources, a Biolog GN2 microplate kit was utilized to analyze and compare the carbon utilization difference between the wild type and the mutant strain. This kit employs a novel and patented redox chemistry. If the strain can utilize a compound, it will develop a color which can be captured by plate reader. Totally 95 carbon sources generally metabolized by aerobic gram negative strain were tested. From the results (Table 1), we confirmed that wild type P17 do utilize a wide range of carbon source, among 95 tested compounds, it is able to utilize 43 compounds. For our mutant strain, it is applausive, the mutant even can utilize more compounds, and 53 compounds tested can be metabolized, which includes 21 organic acids, 22 amino acids, 9 carbohydrates and 11 other compounds (Table 1). These compounds make up a crucial part of the labile materials in the natural water. We also found out that 35 compounds were utilized by both strains. These data indicated that the GFP-P17 mutant had wider range of carbon source utilization pattern as compared with wild type P17.

**Table 1:** Comparison of carbon substrate utilization pattern of GFP-P17 and P17.

	Amino acids	Carbohydrates and Alcohols	Organic acids	others
Used by P17 only		Gentiobiose Latulose Turanose Maltose	α-Keto butyric acid α-Hydroxy butyric Acid	Dextrin
Used by GFP-P17 only	L-Histidine Hydroxy-L-Proline L-Asparagine	D-Galactose D-Trehalose	P-Hydroxy Phenylacetic Acid Urocanic Acid Cis-Aconitic Acid Itaconic Acid Citric Acid D-Gluconic Acid D-Glucosaminic Acid Quinic Acid	Inosine Uridine D-Galactonic Acid Lactone m-Inositol Glycerol
Used by GFP-P17 and P17	L-Alaninamide D-Alanine L-Alanine L-Proline L-Alanyl-glycine L-Pyroglutamic acid N-Acetyl-D-L-Aspartic acid L-Serine L-Glutamic acid	D-Fructose L-Rhamnose Sucrose Xylitol D-Arabitol D-Mannitol D-Mannose	Acetic acid Bromo succinic acid Α-Keto glutaric acid D, L-Lactic acid Malonic acid Propionic acid Β-Hydroxy butyric acid Sebacic acid Glycyl-L-aspartic acid Succinic acid Glycyl-glutamic acid γ-Amino butyric acid Methyl-Pyruate	Glucuronamide Tween 40 Tween 80 D, L-α-Glycerol Phosphate Glucosamine

### Yield factor values of GFP-P17 for AOC determination by flow cytometry

**Fig 4:**
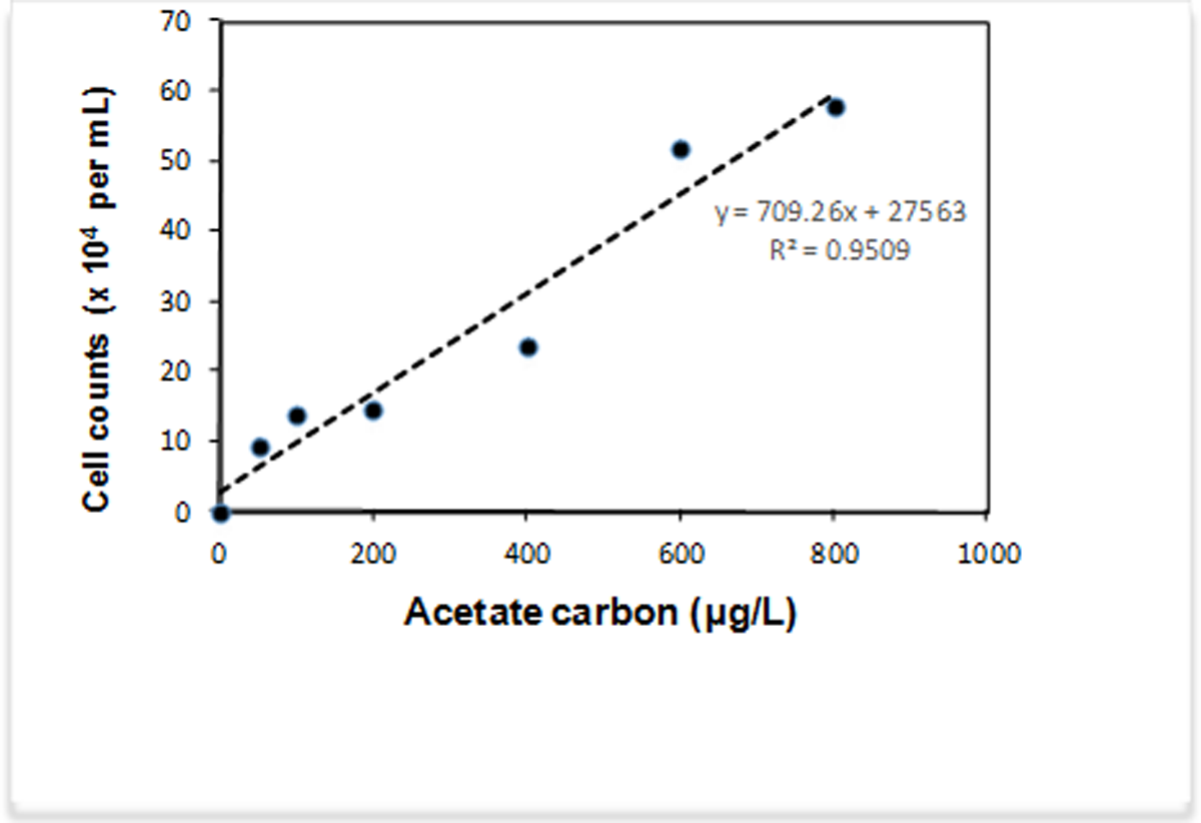
Cell counts for growth of GFP-P17 cells on a defined carbon source (sodium acetate).

After obtaining and characterization of the GFP-P17, we next tested the capability of flow cytometry method to enumerate GFP-P17 growth in standard acetate solution and draw standard curve of P17 cells correlation with different concentration of sodium acetate. Basically, the cells was inoculated in 0–800 µg/L acetate carbon solution and cultured for 3 days prior to flow cytometry analysis. In flow cytometry analysis, bacteria suspension was analyzed directly without staining. As shown in the Fig 4, counts from the FC methods GFP-P17 were all linearly correlated with acetate carbon concentration with a R2 of 0.95. The yield factor of GFP-P17 is 7.1 x 10^5^ cells/µg-C mL.

### Measurement of AOC in water treatment system by flow cytometry method

In a local water works, due to the concerns on some emerging contaminants like PPCP (Pharmaceutical & Personal Care Products), Off-flavour Compounds (MIB, geosmin, etc) in raw water, advanced oxidization technologies (like ozonation) have been introduced for destroying/removing those organic compounds. However, such treatments have resulted in another issue. The broken-down small organic compounds (like AOC) can’t be filtered/removed by traditional sand-filtration during water treatment. AOC can cause bio-stability issue in downstream network, meanwhile may also generate some disinfection-by-products during chlorination treatment. Thus, GAC/BAC filter has to be used in combination with ozonation treatment to remove AOC. Therefore, AOC detection is very critical for water treatment efficiency, particularly for GAC/BAC efficiency/condition monitoring. As shown in the above, cell counts of GFP-P17 was linearly correlated with acetate carbon concentration, indicating the possible utilization of flow cytometry method to detect AOC levels in water samples. Therefore, we next chose water samples from three locations (ozonation inlet, ozonation outlet and GAC outlet) from above mentioned water works and inoculated GFP-P17 to examine AOC concentration. As shown in the Table 2, the AOC concentration in ozonation inlet, ozonation outlet and GAC outlet is about 149, 288 and 187 µg/L detected by non-stained GFP-P17 flow cytometry method. Comparison between this FC method and conventional SPC methods results in a p-value of 0.073, indicating that the data of the two methods are not significantly different. According to the past AOC analysis work by SPC method, there is significantly increased AOC level in ozonation outlet compared to ozonation inlet. And the AOC level decreased after GAC treatment. Consistently, our result shows that the AOC level detected using non-stain GFP-P17 flow cytometry method increases after water ozonation and decreases with GAC removal. The trending of AOC level in the water treatment process detected by this method is similar to that with conventional method although the absolute AOC value may not be exactly the same.

**Table 2:** AOC concentration measured using flow cytometry (FC) method and spread plate counting method(SPC).

Sample No.	Location	AOC level (µg-C/L) Determined by FC with GFP-P17	AOC level (µg-C/L) Determined by SPC
1	Ozonation inlet	149	112
2	Ozonation outlet	288	178
3	GAC outlet	187	111

## Discussion

Although AOC assay was of importance and relevant in determining water quality and biological stability of drinking water, it is not widely used and applicable in practice due to the time consuming and tedious nature of the conventional AOC assay. In last decade, a new strategy to determine AOC level using flow cytometry was proposed by Egli group, which opened a new direction to solve this issue[26]. The time of cell enumeration can be reduced to 15 minutes from 2–3 days compared to spread plate counting. Moreover, the flow cytometry method could easily be automated. The significant disadvantage of this method may be caused by the expense equipment investigate for flow cytometer and additional cells staining procedure. However, one recent study demonstrated that the cost is equal to or lower than those of SPC if the water testing samples reaches 15 per day pending on labour costs and preferred instruments [24]. To further reduce the cost for flow cytometry method, here we explore the possibility to save the staining dye and the time related to the staining.

Strain fluorescence dye staining is a general strategy to reduce water matrix background interfere and machine noise since cell enumeration without staining would overestimate AOC level detected by flow cytometry[28]. In this study, we successfully obtain one GFP tagged P17 strain, which do not need additional staining and makes the flow cytometry method more convenient and more time saving. This Tn5 mediated fluorescent mutant strain is reminiscent of bioluminescent strains obtained by LeChevallier group[23]. The researchers measures the peak bioluminescence and correlate it to AOC concentration. This bioluminescence method is not directly based on cell enumeration as compared to standard method. Our method based on flow cytometry and GFP tagged strain keeps the same rational as standard method for AOC measurement and improves the enumeration efficiency. In this work, we also further analyzed the substrate utilization pattern for our GFP tagged strain. From Biolog analysis, our mutant strain even has the ability to metabolize more low molecular compounds compare to the wild type strain. Broader substrate utilization pattern brings added value for better reflecting AOC in water.

GFP-P17 grew to stationary phase at day 3 in our testing conditions, which is much shorter than that of the wild type strain which needs more than 5 days. This merit also help cut the total AOC measurement time to just 3 days from more than 10 days using SPC method. We also found that GFP-P17 has lower growth yield factor value than other reported strains and methods. This is attributed to the different experimental conditions like temperature, water quality and strains. From our validation assays, however, our method performs as well as the SPC method. This was accomplished through a concurrent plate count assay of the flow cytometry tests shown in Table 2. Standard t tests of SPC and our methods derived AOC values revealed no significant differences for all three types of samples from drinking water treatment processes.

**Fig 5:**
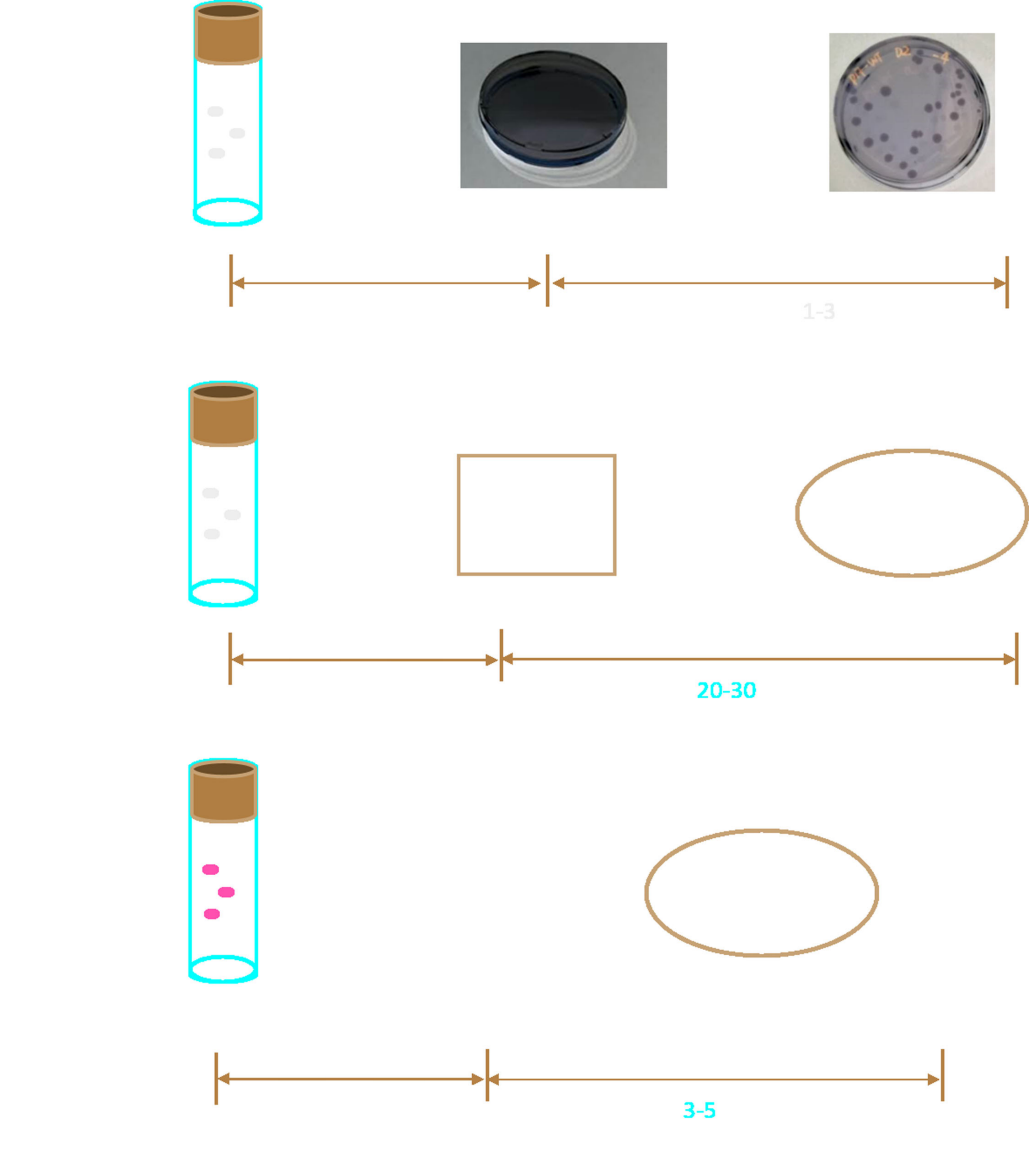
Schematic presentation of our method compared to other methods. A. Conventional method; B, Flow cytometric method with cell staining. C, Our flow cytometric method without cell staining.

Taken together, these study describe a rapid and accurate AOC assay based on newly obtained GFP tagged P17 strain It is a further development of flow cytometry in water AOC measurement with the advantage of avoiding staining procedure(Fig 5). This will be helpful for automation of AOC measurement by flow cytometry. In addition, this GFP-tagged strain has boarder substrate utilization pattern and faster growth in comparison to wild type P17.
