# Correction: Assimilable organic carbon (AOC) determination using GFP-tagged *Pseudomonas fluorescens* P-17 in water by flow cytometry

**DOI:** 10.1371/journal.pone.0209721

**Published:** 2018-12-20

**Authors:** Peng Tang, Jie Wu, Hou Liu, Youcai Liu, Xingding Zhou

There is an error in Fig 5. Please see the correct Fig 5 here.

**Fig 5:**
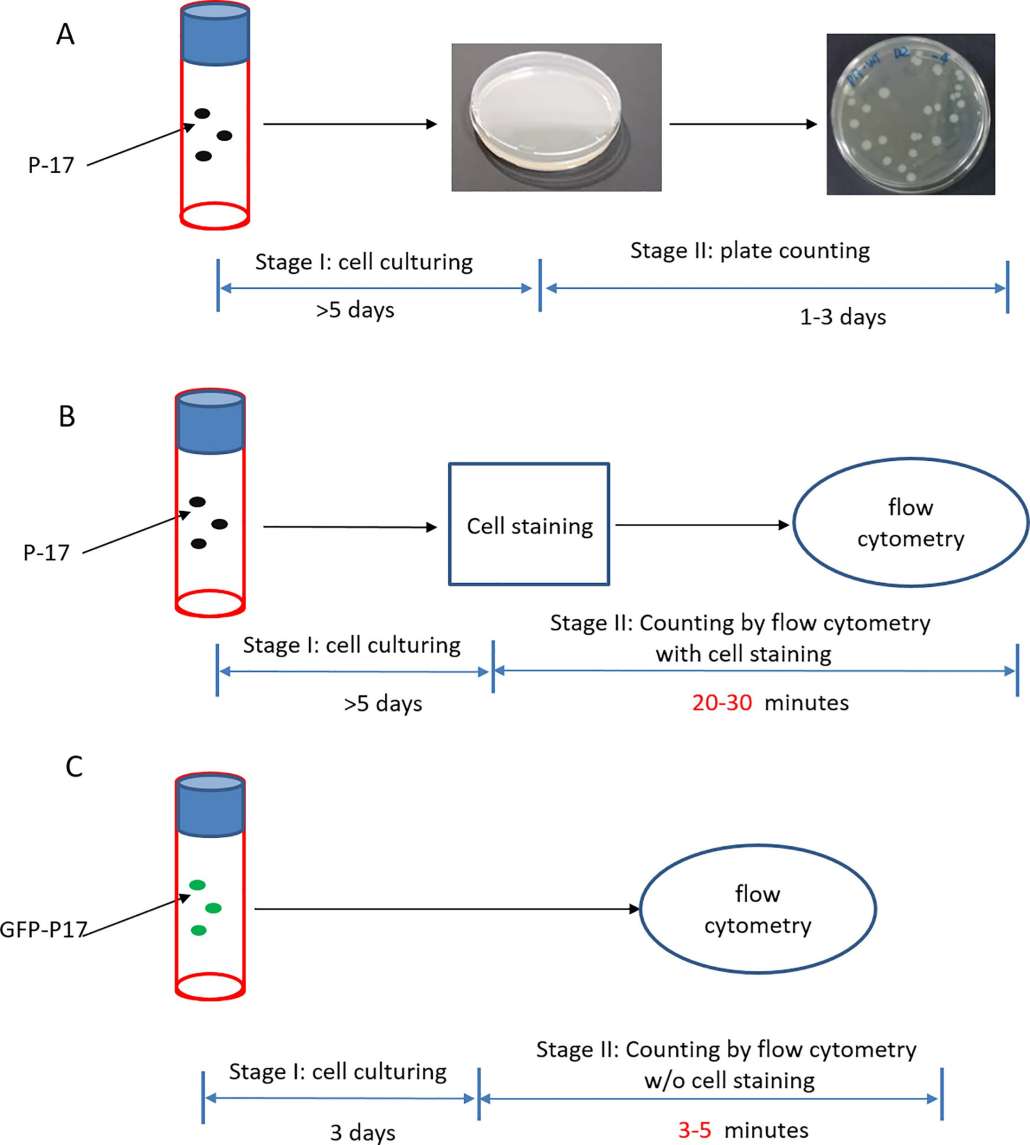
Schematic presentation of our method compared to other methods. **(**A) Conventional method. (B) Flow cytometric method with cell staining. (C) Our flow cytometric method without cell staining.
